# A novel m.7539C>T point mutation in the mt-tRNA^Asp^ gene associated with multisystemic mitochondrial disease

**DOI:** 10.1016/j.nmd.2014.09.008

**Published:** 2015-01

**Authors:** Diana Lehmann, Kathrin Schubert, Pushpa R. Joshi, Karen Baty, Emma L. Blakely, Stephan Zierz, Robert W. Taylor, Marcus Deschauer

**Affiliations:** a Department of Neurology, University of Halle-Wittenberg, Ernst-Grube-Str. 40, Halle/Saale 06097, Germany; b Wellcome Trust Centre for Mitochondrial Research, Institute of Neuroscience, The Medical School, Newcastle University, Framlington Place, Newcastle upon Tyne, UK

**Keywords:** Mitochondria, Multisystemic disease, tRNA^Asp^

## Abstract

Mitochondrial transfer RNA (mt-tRNA) mutations are the commonest sub-type of mitochondrial (mtDNA) mutations associated with human disease. We report a patient with multisytemic disease characterised by myopathy, spinal ataxia, sensorineural hearing loss, cataract and cognitive impairment in whom a novel m.7539C>T mt-tRNA^Asp^ transition was identified. Muscle biopsy revealed extensive histopathological findings including cytochrome *c* oxidase (COX)-deficient fibres. Pyrosequencing confirmed mtDNA heteroplasmy for the mutation whilst single muscle fibre segregation studies revealed statistically significant higher mutation loads in COX-deficient fibres than in COX-positive fibres. Absence from control databases, hierarchical mt-tRNA mutation segregation within tissues, and occurrence at conserved sequence positions, further confirm this novel mt-tRNA mutation to be pathogenic. To date only three mt-tRNA^Asp^ gene mutations have been described with clear evidence of pathogenicity. The novel m.7539C>T mt-tRNA^Asp^ gene mutation extends the spectrum of pathogenic mutations in this gene, further supporting the notion that mt-tRNA^Asp^ gene mutations are associated with multisystemic disease presentations.

## Highlights

• Identification of a novel m.7539C > T mutation in the mt-tRNA^Asp^ gene.
• Confirmation of pathogenicity by single muscle fibre segregation studies.
• Extension of spectrum of pathogenic mutations in the mt-tRNA^Asp^ gene.
• Affirmation of association of mutations in this gene with multisystemic disease.

## 1 Introduction

Mitochondrial diseases are associated with a wide range of different clinical phenotypes, from mild to severe. Diagnosis is difficult if no classical syndrome is present [1]. Some well-characterised, heteroplasmic mitochondrial DNA (mtDNA) mutations associated with specific clinical phenotypes (e.g. m.3243A > G MELAS and m.8344A > G MERRF) are routinely screened, although > 300 different pathogenic mt-tRNA mutations have been described exhibiting marked clinical heterogeneity and hereditability, and are only identified following sequence analysis of the entire 16.6 kb mitochondrial genome [2,3]. Assigning pathogenicity to novel mt-tRNA variants is very important particularly regarding the highly polymorphic nature of mtDNA [4].

A pathogenic heteroplasmic mtDNA mutation has to exceed a certain mutation level within a cell or tissue to cause a disease phenotype [5]. This threshold level varies for each mutation and tissue and is dependent on the OXPHOS metabolism of the tissue [6]. Here we report on a 51-year-old woman, who presented with myopathy, spinal ataxia, deafness, cataract and cognitive impairment, due to a new heteroplasmic point mutation in the mt-tRNA^Asp^ gene.

## 2 Patient and methods

### 2.1 Case report

A 51-year-old woman presented with a one-year history of muscle weakness of arms and legs and intermittent muscle pain in the right thigh. The patient had bilateral hearing loss and had worn a hearing aid for 10 years in her left ear. She had undergone cataract surgery on both eyes at the age of 47. She also complained of intermittent dysphagia and lack of concentration, although a history of seizures was not noted. Family history was unremarkable; her mother developed dementia at a higher age, whilst her 21 year old daughter was healthy.

**Fig. 1:**
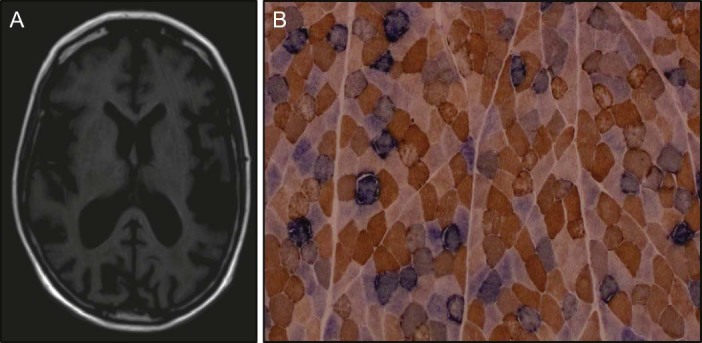
MRI and histochemical findings: (A) cMRI (t1) showing generalised brain volume reduction. (B) Histochemical demonstration of sequential COX and SDH activities revealing numerous COX-deficient (blue reaction product) fibres and evidence of subsarcolemmal mitochondrial proliferation.

Neurological examination revealed pathological laughter and crying, dysarthric speech, proximal accentuated paresis (MRC 4/5), hammer toes and talipes cavus. Arm deep tendon reflex zones were broadened with exhaustible ankle clonus on both sides. Pallhypesthesia of the lower distal extremity has been examined. Romberg test revealed loss of stand. Unterberger's test showed undirected falling tendency. Electroencephalogram revealed multifocal reliable signs of increased cerebral excitability. Needle electromyogram of the brachioradialis muscle revealed distinctive myopathic changes and nerve conduction studies of the tibialis nerve showed an indication for a mixed motoric neuropathy. Sensory neurography was normal. Audiogram revealed severe bilateral inner ear hearing loss on both sides. Ophthalmologic examination showed a regenerative post-cataract on both eyes. Neuropsychological testing revealed severely restricted information processing and instructional understanding. Minimental state examination, however, showed normal results. cMRI showed generalised brain volume reduction (Fig. 1A). Resting lactate levels were normal but mildly elevated in a validated bicycle exercise test (after 10 minutes cycling on 30 Watt 3.9 mmol/l, normal: <2.0) [7]. Creatine kinase was elevated up to 15.2 µmol/l (normal: <2.4) in multiple samples taken at different time points.

### 2.2 Histopathology, biochemistry and molecular genetic studies

Standard histopathological analysis of a muscle biopsy from the biceps brachii muscle was performed. Activities of respiratory chain complexes were determined spectrophotometrically [8]. Total DNA from all available tissue (muscle, urinary epithelia, buccal epithelia, hair shafts, and blood) was extracted by standard procedures; tissues from maternally-related family members were, unfortunately, unavailable. Long-range PCR of muscle DNA was undertaken to detect large-scale rearrangement of mtDNA [9], followed by sequencing of the entire mitochondrial genome in this tissue [10]. Analysis of mtDNA heteroplasmy was carried out by quantitative pyrosequencing including segregation studies within individual cytochrome *c* oxidase (COX)-positive and COX-deficient fibres. The PyromarkQ24 Assay Design Software v.2.0 (Qiagen, Crawley, West Sussex, UK) was used to design locus-specific PCR and pyrosequencing primers for the m.7539C>T mutation (GenBank reference number NC_012920.1). Pyrosequencing was performed on the Pyromark Q24 platform according to the manufacturer's protocol. Quantification of m.7539C>T heteroplasmy levels was determined using Pyromark Q24 software to directly compare the relevant peak heights of both the wild-type and mutant nucleotides at this position [11].

## 3 Results

Muscle biopsy analysis revealed numerous COX-deficient fibres (25% of the total biopsy) and COX-intermediate reacting fibres (25%) in addition to ragged-red-fibres and subsarcolemmal mitochondrial accumulation (5% of all fibres) (Fig. 1B). Biochemical analysis showed decreased activity of respiratory chain complex IV in the patient's muscle (Table 1).

**Fig. 2:**
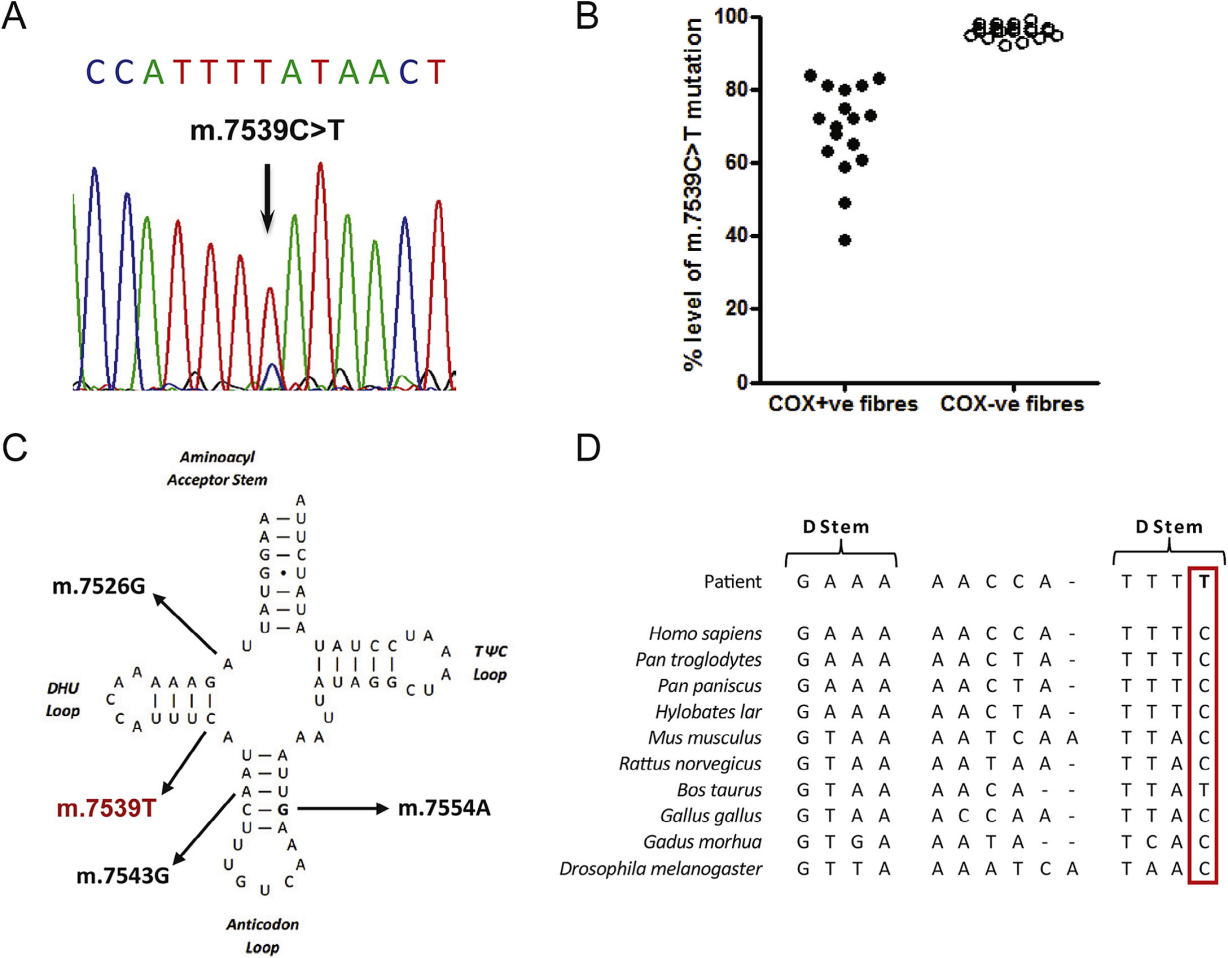
Molecular genetic investigation of patient muscle: (A) sequencing electropherogram demonstrating the heteroplasmic m.7539C>T transition detected in patient muscle. (B) Single fibre PCR analysis clearly shows a marked segregation of the m.7539C>T mutation with a biochemical defect in individual COX-deficient muscle fibres (n = 21) which harbour higher levels of mutation than COX-positive fibres (n = 17). (C) Schematic representation of the mt-RNA^Asp^ cloverleaf structure, illustrating the localisation of the m.7539C>T mutation in the stem of the DHU arm three other known mutations. (D) Phylogenetic conservation of this region of the mt-tRNA^Asp^ gene sequence indicates the mutation affects an evolutionary conserved residue.

Long-range PCR showed no large-scale deletions of mtDNA, prompting sequencing of the entire mitochondrial genome in muscle revealing a novel mutation in the mt-tRNA^Asp^ (*MTTD*) gene – m.7539C>T (Fig. 2A). The highest mutation load level was found in muscle (85% levels of mtDNA heteroplasmy), with lower levels present in urinary epithelial sediment (27%), buccal epithelial cells (15%), hair shafts (10%) and blood (8%), consistent with the segregation pattern of a pathogenic mtDNA mutation. Single muscle fibre analysis of individual COX-positive and COX-deficient fibres detected a statistically-significant higher mutation load in COX-deficient fibres (96.05 ± 0.38 (n = 21)) than in COX-positive fibres (69.12 ± 2.98 (n = 17), p < 0.0001), confirming high levels of the m.7539C>T mutation were associated with a respiratory-deficient phenotype (Fig. 2B).

## 4 Discussion

The phenotype of our patient was characterised by a multisytemic disease presentation with myopathy, spinal ataxia, deafness, cataract and cognitive deficit. These symptoms do not fit with a distinct mitochondrial syndrome such as MELAS or MERRF, but affection of muscle and central nerve system together with inner ear is highly indicative of a mitochondrial aetiology. Sensorineural hearing loss is a common symptom of mitochondrial disease associated with mt-tRNA mutations; cataracts are reported in single patients only (e.g. reported pathogenic m.14685G > A, m.12264C > T, m.1606G > A and m.3274A > G mutations) [12].

The clinical picture together with the histopathogical findings characterised by focal COX deficiency and mitochondrial proliferation prompted us to perform sequencing of the mitochondrial genome leading to the identification of a novel heteroplasmic mt-tRNA point mutation. The pathogenicity of the m.7539C>T mutation is unequivocally proven according to accepted criteria published by Yarham et al. [13]. First, it is not listed as a SNP on publically-available databases of common mtDNA variants including MitoMAP (http://www.mitomap.org/MITOMAP) or the Human Mitochondrial Genome Database (http://www.mtdb.igp.uu.se/index.html) and we have not detected this variant amongst > 980 in-house human mtDNA sequences. Second, the m.7539C>T mutation is heteroplasmic and located at a conserved position – within the DHU-stem of the mt-tRNA^Asp^ – leading to the disruption of a relatively evolutionary-conserved base pair (Fig. 2C and D). The mutation is present at highest levels in the patient's muscle, a clinically-affected tissue, whilst single muscle fibre analysis clearly demonstrates that the mutation segregates with COX-deficiency. The mutation showed a very high threshold in muscle suggesting a rather mild functional effect of the mutation. In urinary epithelial cells there was a higher level of heteroplasmy of the m.7539C>T mutation compared to blood as seen in other mtDNA tRNA mutations. However, the relative proportions of mtDNA heteroplasmy observed in our patient confirm that muscle remains the tissue of choice for Sanger sequencing of the mitochondrial genome although this would represent less of a concern with next-generation sequencing protocols. The low level of heteroplasmy in blood makes it more likely that the mutation is sporadic and is not transmitted [14]. Unfortunately our patient's mother and clinically-unaffected daughter declined genetic testing so we are unable to determine whether the mutation within this family has arisen de novo or exhibits a maternal transmission pattern.

Given the large repertoire of reported mt-tRNA mutations, it is perhaps surprising that to date only three *MTTD* gene mutations have been described with clear evidence of pathogenicity. These include a m.7526A > G transition associated with exercise intolerance [15], a m.7543A > G mutation leading to myoclonic seizures, developmental delay, and severe behavioural problems [16] and a m.7554G > A transition associated with a multisystemic disease presentation comprising myopathy, ataxia, nystagmus, and migraine [17]. In conclusion, the novel m.7539C>T mt-tRNA^Asp^ gene mutation extends the spectrum of pathogenic mutations in this gene, further supporting the notion that mt-tRNA^Asp^ gene mutations are associated with multisystemic disease presentations.

## Funding

DL, PRJ, SZ, and MD are members of the German mitoNET funded by the German Ministry of Education and Research. RWT receives support from the Wellcome Trust Centre for Mitochondrial Research (096919Z/11/Z), the Medical Research Council (UK) Centre for Translational Muscle Disease research (G0601943), the Lily Foundation and the UK NHS Highly Specialised “Rare Mitochondrial Disorders of Adults and Children” Service.

## Authors' contributionss

DL: Experimental work, preparation of manuscript.

KS: Experimental work.

PRJ: Experimental work.

KB: Experimental work.

ELB: Interpretation of the data and critical review.

SZ: Interpretation of the data and critical review.

RWT: Concept, supervision, interpretation of the data and critical review.

MD: Concept, supervision, interpretation of the data and critical review.

## Figures and Tables

**Table 1:** Enzyme activity of respiratory chain complexes showing decreased activity of respiratory chain complex IV in patient muscle.

Respiratory chain complexes	Enzyme activity (U/g tissue)	
	Patient	Controls (n = 20) mean ± SD [range]
Complex I	0.44	0.9 ± 0.6 [0.35–2.5]
Complexes II + III	1.0	1.8 ± 0.8 [0.8–2.6]
Complex IV (COX)	1.6	6.3 ± 1.5 [4.5–9.3]
Citrate synthase	4.9	8.4 ± 2.7 [4.0–11.2]
